# Capturing the dynamic integrity of carbocyanine fluorophore-based lipid nanoparticles using the FRET technique†

**DOI:** 10.1039/d4tb02653e

**Published:** 2025-01-22

**Authors:** Siyu Long, David A. Turner, Kevin J. Hamill, Louise S. Natrajan, Tom O. McDonald

**Affiliations:** a Department of Chemistry, University of Manchester Oxford Road Manchester M13 9PL UK Thomas.mcdonald@manchester.ac.uk; b Institute of Life Course and Medical Sciences, University of Liverpool 6 West Derby Street Liverpool L7 8TX UK; c Department of Materials, The University of Manchester Oxford Road Manchester M13 9PL UK; d Henry Royce Institute, The University of Manchester Oxford Road Manchester UK

## Abstract

Nanoparticles capable of dynamically reporting their structural integrity in real-time are a powerful tool to guide the design of drug delivery technologies. Lipid nanoparticles (LNPs) offer multiple important advantages for drug delivery, including stability, protection of active substances, and sustained release capabilities. However, tracking their structural integrity and dynamic behaviour in complex biological environments remains challenging. Here, we report the development of a Förster resonance energy transfer (FRET)-enabled LNP platform that achieves unprecedented sensitivity and precision in monitoring nanoparticle disintegration. The FRET-based LNPs were prepared using nanoprecipitation, encapsulating high levels of 3,3′-dioctadecyloxacarbocyanine perchlorate (DiO) and 1,1′-dioctadecyl-3,3,3′,3′-tetramethylindocarbocyanine perchlorate (DiI) fluorophores as the donor and acceptors, respectively. The resulting LNPs had a mean diameter of 114 ± 19 nm with a distinct FRET signal. An optimal energy transfer efficiency of 0.98 and an emission quantum yield of 0.13 were achieved at 11.1% fluorophore loading in the LNPs, balancing efficient energy transfer and minimal aggregation-induced quenching. Using the FRET reporting, three dissociation stages of FRET LNPs were observed: solvation, indicated by an increased emission intensity; swelling and partial dissolution, evidenced by changes in emission maxima and mean size; and complete dissociation, confirmed by emission solely from DiO and the absence of particles. Testing the nanoparticles in live cells (telomerase-immortalised human corneal epithelial cells, hTCEpi cells) revealed a direct link to the disappearance of the FRET signal with the dissociation of FRET NPs. The nanoparticles initially exhibited a strong extracellular FRET signal, which diminished after cellular internalisation. This suggests that the LNPs disintegrate after entering the cells. These findings establish FRET-based LNPs as a robust tool for real-time nanoparticle tracking, offering insights into their integrity and release mechanisms, with potential applications in advanced drug delivery and diagnostics.

## Abstract 2

Nanoparticles capable of dynamically reporting their structural integrity in real-time are a powerful tool to guide the design of drug delivery technologies.

## Graphical abstract

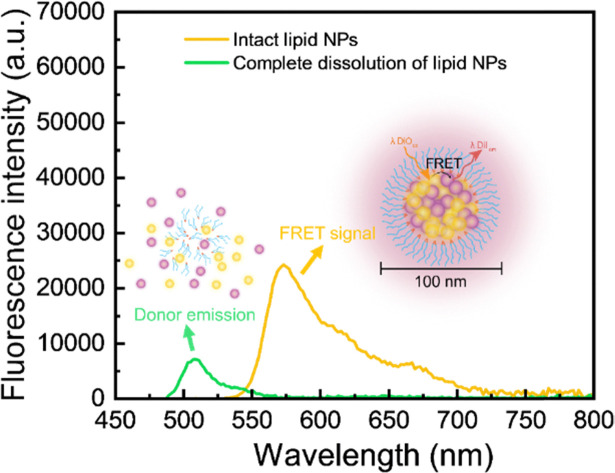

## Introduction

Lipid nanoparticles (LNPs) are widely recognised as a pivotal technology in drug delivery due to their ability to protect active pharmaceutical ingredients (APIs) against degradation and enable sustained release.^1,2^ LNPs have gained prominence for the delivery of nucleic acid,^3^ particularly mRNA.^4,5^ Beyond this, LNPs can encapsulate a diverse range of actives, including small molecules,^6–9^ biologics,^10^ and diagnostic labels.^11^ For example, the incorporation of fluorescent labels enables precise cellular tracking and LNPs can help solve fluorescence problems commonly observed in small molecule labelling by creating a microenvironment that quenches reactive oxygen species^12^ and other photochemical reactions responsible for photobleaching and photodegradation.^13^ However, understanding the dynamic behaviour of LNPs, such as structural integrity and dissociation in biological environments, remains a significant challenge in advancing their practical applications.

Tracking LNPs provides the knowledge of their *in vivo* behaviours (pharmacokinetics, distribution, degradation, elimination, *etc.*). For nanocarriers, several strategies for particle detection and tracking have been demonstrated focussing on collecting physical signals from particles or their components.^14^ These typically utilise isotopes^15^ or fluorophores^16,17^ as labels entrapped within the particles. These strategies require that probes remain unreleased, and particles cannot degrade in biological environments for accurate representation. If the particle components are non-biodegradable and the labels remain associated with the particles, then such strategies can enable precise tracking of the particles. However, in the case of LNPs, where the main constituents of the particles are biodegradable lipids such as including triglycerides, diglycerides and monoglycerides^18,19^ means that the labels can be released from the particles in a biological environment. When this happens, the free or released probes still emit signals, making it difficult to distinguish particle-labelled signals from these non-labelled signals.^20^ One strategy to address this involves monitoring nanocarriers through the formation of fluorophore aggregates.^20–22^ For example, fluorescent probes dipyrrometheneboron difluoride fluorophores (BODIPY)^20^ were simultaneously trapped into LNPs, giving rise to fluorescence signals. When the fluorophores escaped from the LNPs into the surrounding aqueous environment, fluorophores aggregated and quenching was detected, thus inferring LNP degradation. Yet, this work did not provide information on dynamic changes in particle structures, focusing instead on the release of the fluorophore upon particle dissociation.

To address this, we consider here the use of Förster resonance energy transfer (FRET) in this work. FRET is a non-invasive fluorescence technique in which an excited state fluorophore (the donor) transfers its energy to another molecule (the acceptor). The efficiency of this energy transfer is highly distance-dependent, typically occurring over a range of 1–10 nm, making FRET a powerful tool for monitoring nanoscale interactions. By observing FRET emission behaviour, it is possible to obtain accurate distance information between the two upon nanocarrier degradation/API delivery.^23^ The primary consideration in designing an effective FRET system is the careful selection of donor and acceptor fluorophores. The spectral overlap between the absorbance of an acceptor and emission of a donor of more than 30% is required.^24^ In addition, the Förster distance (*R*_0_) between these two fluorophores need to be less than 10 nm for FRET to occur.^25^ The dissolution of FRET labelled nanoparticles is reported as a loss of FRET signals as the separation between the two fluorophores is increased upon dissolution.^26,27^ Thus, a FRET labelling approach enables real-time monitoring of not only the location of nanoparticles but also reporting on changes in the integrity of particles based on fluorescence. A FRET labelling approach has been conducted in various non-lipid NPs, including inorganic NPs,^28,29^ nanosuspensions,^30^ micelles^31,32^ and polymeric NPs.^33–36^ Genovese *et al.*^28^ used a coumarin as a donor molecule to transfer energy to a BODIPY derivative fluorophore as the acceptor molecule to avoid intermolecular quenching in silica NPs, optimising the FRET efficiency to 0.91 and enhancing the molar absorption coefficient and quantum yield of coumarin. Delledonne *et al.*^31^ studied the FRET efficiency in cetyltrimethylammonium bromide (CTAB) micelles together loaded with indocarbocyanines, including 1,1′-dioctadecyl-3,3,3′,3′-tetramethylindocarbocyanine perchlorate (DiI), 1,1′-dioctadecyl-3,3,3′,3′-tetramethylindodicarbocyanine perchlorate (DiD) at various CTAB concentrations with both below and above the critical micellar concentration (CMC). In addition, numerous studies have reported on the kinetics of drug release^33^ and the integrity^34–36^ of polymeric NPs using the FRET technique. To the best of our knowledge, there are only a few papers that have reported FRET-based LNPs.^37–41^ These studies focussed on the use of the FRET reporting to monitor payload release, stability and biological accumulation, or sensing but did not investigate how the dissolution behaviour of the LNPs is related to the FRET emission behaviour. Additionally, the only study that quantified the FRET ratio found a FRET efficacy of 80%,^37^ which will likely limit the sensitivity of the particles to reporting changes that occur in the particles. Therefore, while fluorescence-based techniques, such as FRET, have been explored for nanoparticle tracking, they typically suffer from low fluorophore loadings, low FRET efficacy, aggregation-induced quenching, or an inability to correlate fluorescence signals with real-time structural changes. Existing studies often focus on end-point measurements, leaving a critical gap in understanding the dynamic integrity of LNPs during degradation or cellular uptake.

In this work, we introduce a high-loading FRET-based LNP system that uniquely couples the high encapsulation efficiency of fluorophores with real-time fluorescence tracking. By achieving high energy transfer efficiency while minimising aggregation-induced quenching, this system enables the monitoring of three distinct stages of nanoparticle dissociation, being swelling, partial disintegration, and complete disintegration. To do this, FRET-based LNPs were prepared by nanoprecipitation using DiO (donor) and DiI (acceptor) fluorophores as the FRET pair. We studied the effect of increased fluorophores on the size distribution of NPs by dynamic light scattering (DLS) measurement and determined an optimum fluorophore loading that favoured an efficient FRET compromised with a minimum self-quenching of the LNPs by spectrometer measurement. Then we investigated the role of self-quenching at high local fluorophores in LNPs and selected a formulation that provided a balance of the FRET signal and emission intensity. Finally, the dissolution process of LNPs was monitored by the incremental addition of DMSO. The FRET signal was observed, suggesting that particles are integrated to allow for the proximity of the FRET pair, while a gradual decrease in the FRET signal and an increase in donor molecule emission show that the particles were dissolving. This work provides a key tool to address key challenges in tracking nanoparticle integrity. The ability to monitor the dynamic integrity and dissolution behaviour of LNPs would be highly valuable as the integrity of LNPs influences their functionality, particularly in biological applications such as targeted delivery or controlled release.^42^

## Materials and methods

### Materials

DiI; DiIC18(3) (1,1′-dioctadecyl-3,3,3′,3′-tetramethylindocarbocyanine perchlorate) (Thermo Fisher Scientific), DiO; DiOC18(3) (3,3′-dioctadecyloxacarbocyanine perchlorate) (Thermo Fisher Scientific), DMSO (dimethyl sulfoxide, HPLC grade, 99.9+%, Thermo Fisher Scientific), tricaprin (TCI-UK), DI water, poloxamer-188 (Cambridge Bioscience Limited), Rhodamine 101 inner salt (Sigma-Aldrich), Fluorescein (CAS no. 2321-07-5, Sigma-Aldrich), ethanol (absolute, > = 99.8%, Thermo Fisher Scientific), NaCl (Thermo Fisher Scientific), and dialysis tubing cellulose membranes (molecular weight cut-off 14 000, Merck Life Science UK Limited) were employed in this study. All reagents were used as supplied.

### Synthesis of fluorescence-based lipid nanoparticles

Fluorescence-based LNPs (DiO NPs, DiI NPs and (DiO + DiI) NPs) were fabricated *via* nanoprecipitation. Specifically, the hydrophobic phase was composed of tricaprin (0.06% w/v), fluorophores (DiO or DiI or a mixture of 50% DiO and 50% DiI (*i.e.*, (DiO + DiI)) and 1 mL of DMSO, which were placed in a 4 mL vial under ultrasonication (to ensure a homogeneous organic phase, it was sonicated for 2 h in a bath sonicator (ultrasonic frequency (effective/max): 50/60 Hz; ultrasonic power: 60/240 W; SONOREX DIGITEC DT52, Germany). The weight percentages of fluorophores (3%, 5.9%, 11.1%, 20% and 33.3%) were with respect to tricaprin dissolved in DMSO. The aqueous phases consisting of amphiphilic poloxamer-188 solution in deionised (DI) water with a concentration of 0.1% w/v at room temperature were then gently mixed and injected into the hydrophobic phase under constant stirring with a 10-position digital magnetic hotplate stirrer (RT10, IKA, UK) at 800 rpm. The shot of the lipid solution in DMSO was charged by aspirating the DMSO into a syringe and then clamping this syringe over the aqueous solution and then removing the plunger from the syringe. This approach resulted in the DMSO dripping into the aqueous phase at a consistent rate. Pure LNPs (*i.e.* no fluorophores) were prepared as control samples using the same procedure, except that no fluorophores dissolved in DMSO. For the physical mixture groups (the mixture of DiO NPs and DiI NPs) used as comparison group, each of the fluorescence-based NPs was first prepared using the same procedure mentioned above and then directly mixed them. Groups of samples were dialysed against DI water for 12 h at 4 °C in dialysis membrane bags (molecular weight cutoff [MWCO] = 14 000, Merck, UK) to remove residual DMSO. The quantitative compositions of all formulations are shown in Table S1 (ESI†).

### Characterisation of lipid nanoparticles

#### Dynamic light scattering (DLS)

The size distribution of the NPs was obtained using dynamic light scattering (DLS) measured with a Zetasizer Ultra (Malvern, UK) equipped with an avalanche photodiode detector. All measurements were carried out at a fixed backscattering angle of 173° and fixed measurement position at 4.65. The measurements were averaged for three runs. For the dissolution study, all size distribution graphs were calculated based on the fitting results from experimental viscosity values that were fitted by the 6th grade polynomial.^43^

#### Scanning electron microscopy (SEM)

The sizes of the NPs were estimated from SEM images. All specimens were placed on a silicon wafer with a drop and then allowed to dry. The dried samples were then coated with Au/Pt using a Quorum sputter-coater Q150R Plus (an Au/Pd target 80 : 20 was used at 50 mA current for 8 s) prior to imaging. The morphological characterisation by scanning electron microscopy (SEM) was carried out using a Zeiss ULTRA 55 (EHT = 1.5 kV, WD = 5.5 mm).

#### Spectroscopic properties

The absorbance spectra of all fluorescence formulations were measured by using an ultraviolet-visible spectrometer (UV-vis) Lambda 365 (PerkinElmer, UK). It should be noted that all samples were diluted using the same procedure described above for DLS measurement, considering that the absorbance of the particles, which is directly related to the amount of fluorophore encapsulated, remains below 0.1 to avoid inner filter effects.^25,44^ To identify the photophysical properties of DiO and DiI solutions, DiO and DiI were separately dissolved in DMSO at the same fluorescence concentration as fluorophore NPs(5.9% weight percentage with respect to lipid) mentioned above. The final concentrations for measurement were then diluted with DI water. In particular, DiO(11.1%) solution and DiI(11.1%) solution were each diluted in a 1 : 4 volume ratio of fluorophore solution to DI water.

The fluorescence steady state spectra of all samples were recorded on an FLS1000 spectrophotometer (Edinburgh instruments, UK) equipped with a 450 W steady state xenon lamp (with double 325 mm focal length excitation and emission monochromators in Czerny-Turner configuration), and a red sensitive photomultiplier in Peltier (air cooled) 53 housing (Hamamatsu R928P). All samples were measured using standard disposable cuvettes (polystyrene) with a pathlength of 10 mm at an excitation wavelength of 405 nm. The emission was collected at 510 nm for DiO, 570 nm for DiI and the dual fluorophores (DiO and DiI). The slit widths were set to 2 nm. An optimal excitation wavelength of 405 nm was selected with the consideration of avoiding the direct excitation of DiI (acceptor) (in Fig. S1, ESI†). Relative fluorescence quantum yields (*Φ*_f_) were determined by plotting the integrated emission as a function of absorption at an excitation wavelength (405 nm) of 3–5 serial dilutions and comparing the emission spectra of rohdamine-101 in ethanol (*Φ*_f_ = 1.0, for DiI series).^25,45^

#### Cell culture

Telomerase-immortalised human corneal epithelial cells, hTCEpi cells (RRID: CVCL_AQ44),^46^ were cultured at 37 °C with 5% CO_2_ in keratinocyte-serum-free medium (KSFM) supplemented with bovine pituitary extract (BPE, 0.05 mg mL^−1^), human recombinant epidermal growth factor (5 ng mL^−1^, Thermo Fisher Scientific) and 0.15 mM CaCl_2_ (Sigma-Aldrich). 5 × 10^5^ cells were plated overnight on 35 mm glass-bottomed dishes (MatTek Corporation, Ashland, Massachusetts, USA) and then 100 μL of nanoparticle solution was added to the dish immediately prior to imaging.

For the acceptor photobleaching measurements *in vitro*, FRET was performed using an inverted Zeiss LSM900 with an Airyscan 2 spectral detector mounted on an Axio Observer 7 microscope with a 63× 1.4NA oil-immersion objective (Zeiss). DiO and Dil were excited with a 488 nm laser diode and the emitted light was collected in 8 images separated by 10 nm between 425 and 650 nm in lambda scanning mode. Separation of reference spectra for DiO and Dil was performed using the linear unmixing algorithms of the Zen version 3.8 (Zeiss), using reference spectra from DiO and Dil alone, and the fluorescence spectra were separated into DiO and DiI channels. FRET was assayed by photobleaching the Dil (Acceptor) using 50 iterations of 561 nm laser light with no attenuation from the acousto-optical tuneable filter (AOTF).

## Results and discussion

**Fig. 1:**
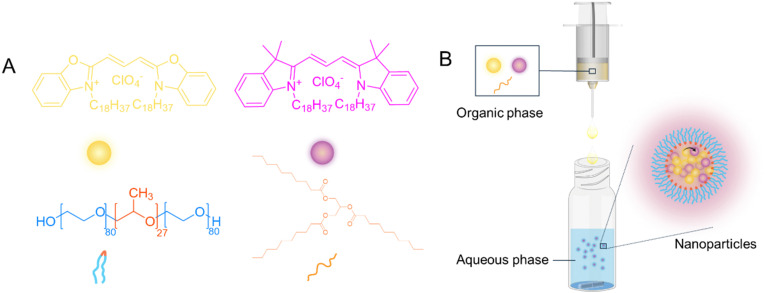
Schematic illustration of the preparation for fluorescence-based lipid nanoparticles. (A) Molecular structures of DiO and DiI (fluorophores), poloxamer-188 (stabiliser) and tricaprin (lipid). (B) Synthesis of FRET-based lipid nanoparticles by nanoprecipitation.

Fluorescence LNPs were produced by a nanoprecipitation method. In this process (shown schematically in Fig. 1), an organic phase containing lipid and fluorophores is added to the aqueous phase containing a stabiliser (the chemical structures of the different components are shown in Fig. 1A). As the solvents mix, a dilution of the organic solvent causes the solute (lipid and fluorophores) to become insoluble, leading to supersaturation, which drives nucleation (Fig. 1B). The nuclei then grow through the incorporation of lipophilic lipid and fluorophores, forming stable particles with the aid of an appropriate stabiliser.^47^ The stabiliser used in this work was poloxamer-188, a non-ionic triblock copolymer composed of a central poly(propylene oxide) (PPO) block flanked by two hydrophilic poly(ethylene oxide) (PEO) blocks and has been extensively studied for LNPs stablisation.^48–54^ It typically contains 80 ethylene oxide units and 27 propylene oxide units (Fig. 1A). The critical micellar concentration (CMC) for poloxamer-188 has been reported to range from 6.9 × 10^−6^ M to 1.2 × 10^−4^ M at the Krafft temperature of 25 °C.^49,55,56^ In this study, a concentration as the bottom of this range, 1.2 × 10^−4^ mM (*i.e.* 0.1 w/v) poloxamer-188 solution was used for the preparation of all NPs. The two fluorophores DiO and DiI are lipophilic fluorophores, due to the presence of two alky chains (C18) on each molecule. Therefore, by including fluorophores in the organic solvent, LNPs encapsulating a combination of two fluorophores, DiO and/or DiI could be obtained.

### Particle properties of LNPs

First, the effect of fluorophore loading on the production of LNPs by nanoprecipitation was investigated. Here FRET-based LNPs with varying loadings were prepared and named as (DiO + DiI)(3%) LNPs, (DiO + DiI)(5.9%) LNPs, (DiO + DiI)(11.1%) LNPs, (DiO + DiI)(20%) LNPs and (DiO + DiI)(33.3%) LNPs, corresponding to the percentage mass of the total fluorophores and the tricaprin (lipid) used to form the particle core. In the case of single fluorophore LNPs, *i.e.* DiO or DiI alone LNPs, each had 5.9% loadings named as DiO(5.9%) LNPs and DiI(5.9%) LNPs. Solutions containing fluorophores with a 11.1% fluorophore loading were named DiO(11.1%) DMSO and DiI(11.1%) DMSO for comparison.

When investigating the effect of fluorophore loading on the formation of LNPs, all five formulations ((DiO + DiI)(3%) LNPs, (DiO + DiI)(5.9%) LNPs, (DiO + DiI)(11.1%) LNPs, (DiO + DiI)(20%) LNPs and (DiO + DiI)(33.3%) LNPs) were successfully achieved, showing that up to 33.3% fluorophore loadings were possible. These loadings made are in a higher range in the lipid matrix, reaching a 20-fold improvement over some existing fluorescence-based lipid nanocarriers.^37,38^

**Fig. 2:**
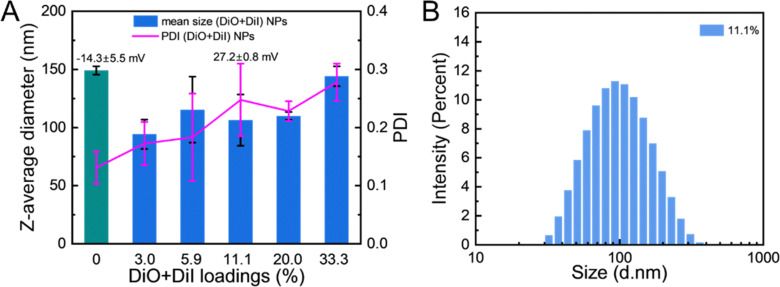
Dual fluorophore loaded LNPs could be prepared at a range of fluorophore loadings. (A) The hydrodynamic diameters and zeta potential of nanoparticles with different (DiO + DiI) loadings. (B) The size distribution of (DiO + DiI)(11.1%) LNPs as measured by DLS.

The particles were characterised by DLS to measure mean diameters and polydispersity index (PDI) values. As shown in Fig. 2A, the formulation without fluorophores exhibited the largest diameter at 149 ± 4 nm and the lowest PDI of 0.13 ± 0.03, indicating a uniform distribution of the pure LNPs. Upon the addition of fluorophores, the diameters of particles decreased to the minimum of 94 ± 13 nm, while the PDI gradually increased to the maximum of 0.28 ± 0.03. The high local fluorophore concentration contributed to an increase of the particle size uniformity. Both DiO and DiI are cationic molecules due to the quaternary ammonium within their structure. The analysis of the FRET-based NPs by electrophoretic mobility showed that the zeta potential of the (DiO + DiI)(11.1%) LNPs possessed a positive zeta potential of 27.2 ± 0.8 mV with all FRET fluorophore containing LNPs also exhibiting positive zeta potential as well, as illustrated in Fig. S2 (ESI†). The positive zeta potential value of the fluorophore containing LNPs was due to the cationic nature of the fluorophore molecules. When compared with the LNP formulation without fluorophores (zeta potential −14.3 mV), some of the fluorophores provided surface charge to the particles. Thus, the colloidal stability of the particles is likely a result of both the steric interaction from poloxamer-188 and the electrostatic repulsion from fluorophores. This additional electrostatic stabilisation provided by the cationic fluorophores may explain why the LNP formulations loaded with fluorophores tended to be smaller in diameter.

The colloidal stability of the formulations was then studied in stability studies carried out both at 5 °C and 37 °C over 24 hours for (DiO + DiI)(11.1%) LNPs. Under both conditions, the (DiO + DiI)(11.1%) LNPs remained stable as indicated by DLS measurement (Fig. S3, ESI†). Moreover, both the LNPs without and with fluorophores showed good stability when stored at 5 °C (as potential storage condition) for 4 weeks (Fig. S4, ESI†), which suggests that the presence or absence of fluorophores had no impact on particle stability. The LNPs formulation with 11.1% fluorophore loading was then selected for analysis by SEM, revealing spherical particles with the diameter of *ca.* 80 nm (ranging from 41 to 111 nm) from the SEM images (Fig. S5, ESI†) and which was similar to that seen in the DLS size distribution (Fig. 2B). The SEM analysis gave smaller particle diameters compared to the values obtained by DLS because DLS measures the hydrodynamic diameter, while SEM measures the particles in the dry form. The remaining size distribution graphs of FRET-based LNPs with different fluorophore loadings are shown in Fig. S6 (ESI†). This analysis showed that fluorophore loaded LNPs could be successfully produced by nanoprecipitation.

### Photophysical properties of NPs

FRET is an energy transfer process between two fluorophores through dipole–dipole coupling and its efficiency is distance-dependent. DiO and DiI are a widely used FRET pair.^33,36,57–59^ As illustrated in Fig. S7 (ESI†), there is an extensive overlap of the spectrum between the emission spectrum of the DiO molecule and the absorption spectrum of the DiI molecule, meeting the critical requirement for the efficient energy transfer.

**Fig. 3:**
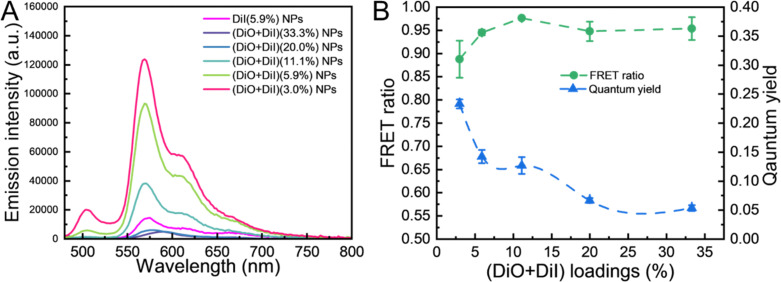
The fluorescence properties of dual fluorophore loaded LNPs. (A) Fluorescence spectra (at an excitation of 405 nm) of (DiO + DiI)(11.1%) LNPs with different loadings and pure DiI(5.9%) LNPs. (B) FRET ratio and quantum yield of (DiO + DiI)(11.1%) LNPs with different loadings.

We first studied the emission spectra of DiO and DiI as a FRET pair together loaded into LNPs under the excitation of the DiO molecule (405 nm). LNPs containing only Dil, DiI(5.9%) LNPs, were prepared as a control sample, with an emission wavelength at 575 nm (Fig. 3A). For the dual-fluorophore (DiO + DiI) LNPs, as the total amount of fluorophores increased, the donor DiO-related emission wavelength (510 nm) exhibited a gradual quenching, indicating FRET occurrence. At 11.1% fluorophore loading, the NPs initially showed a near complete quenching of DiO-related emission (510 nm) combined with a clear emission of the acceptor DiI at 570 nm. Moreover, the DiI acceptor emission signals of the (DiO + DiI)(11.1%) LNPs were threefold higher than those of the DiI(5.9%) LNPs due to energy transfer from DiO to DiI. In addition, for all the formulations, increasing the dual-fluorophore loading resulted in a gradual quenching and a broadening of the DiI-related emission wavelength (∼570 nm). We also found a slight blue shift in the fluorescence of LNPs (∼507 nm for DiO, ∼570 nm for DiI) compared to pure fluorophore solutions (515 nm for DiO, 583 nm for DiI) shown in Fig. S7 (ESI†). Both the significant quenching and blue shift in spectra can be explained by aggregation-induced quenching^60^ as the containment of the fluorophores within the LNPs (diameter ∼100 nm) creates an environment that brings the fluorophores in close proximity. Additionally, it may be possible that within the core of the LNPs the fluorophores partially segregate from the lipid and form H-aggregates in NPs in which at least two fluorophores possess a co-facial alignment,^44,61,62^ resulting in deactivation.

The FRET ratios of the LNPs were then calculated by the semi-quantitative method (*F*_AD_/(*F*_AD_ + *F*_DA_)),^63^ where the *F*_AD_ and *F*_DA_ represent the emission of the acceptor molecule and donor molecule, respectively, upon the excitation of the donor molecule (405 nm). All samples followed the same dilution procedure described in the spectroscopic properties section prior to all measurements. Fig. 3B illustrates the changes in the relative maximum as the overall dual fluorophore loadings increase. The highest FRET ratio of 0.98 was observed at 11.1% (DiO + DiI) loading. In addition, the relative fluorescence quantum yield of fluorophores is the evidence of their efficiency for on-demand light emission. Although the quantum yield decreased with higher loading levels, these values were about three-times greater than those fluorophores in ethanol.^64^ At 11.1% loading, the reduction in the curve began to be modest. We therefore selected the (DiO + DiI)(11.1%) LNPs as the sample for further study because it provides the highest FRET ratio.

The fluorescence stability of (DiO + DiI)(11.1%) LNPs was investigated as well. Over 24 hours, a marginal reduction (0.3% for 37 °C) in the FRET ratio was observed, indicating that the fluorescence of the NPs was well-stabilised under tested temperature conditions (Fig. S8 (ESI†), FRET ratio of (DiO + DiI)(11.1%) NPs at 5 °C and 37 °C over 24 hours.). This confirms that the fluorescence-based LNPs were stable, and the fluorophores did not show any tendency to aggregate once successfully co-located in the LNPs (Fig. S3, ESI†).

**Fig. 4:**
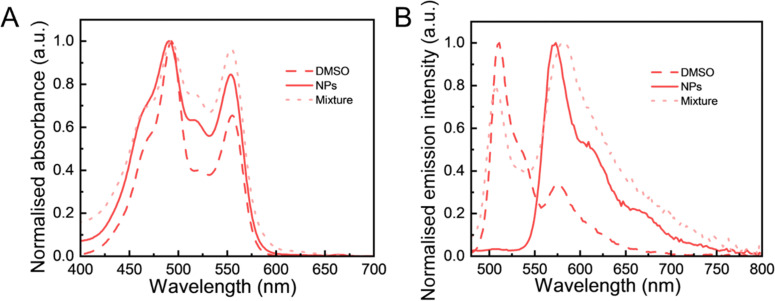
The normalised photophysical properties of different fluorescence samples. (A) Absorbance and (B) fluorescence spectra of (DiO + DiI)(11.1%) DMSO (DMSO), (DiO + DiI)(11.1%) LNPs (NPs) and a physical mixture of DiO(5.9%) LNPs and DiI(5.9%) LNPs (mixture) (at an excitation of 405 nm).

To further study the effect of particle composition on the FRET behaviour, we prepared a mixture of pure DiO(5.9%) LNPs and DiI(5.9%) LNPs for comparison. The reason of choosing these two separate DiO and DiI nanoparticles was to ensure a consistent total fluorophore concentration across all nanoparticle samples. The absorption spectra of the samples were recorded to investigate the formation of aggregates of fluorophores. These spectra reflect the interactions among fluorophores that affect their electronic transitions, providing insights into the molecular environment.^44^ In Fig. 4A, the absorption spectrum of the physical mixture showed a profile similar to that of (DiO + DiI)(11.1%) LNPs with a minor increase of absorbance in the 0–1 vibronic band (at ∼ 523 nm). The similarity of these spectra with those measured in NPs encapsulated with DiO and DiI means that the fluorophores experienced a similar environment in LNPs at the current 11.1% (DiO + DiI) loading. Particularly, DiO-related absorbance (∼490 nm) and DiI-related absorbance (∼553 nm) of (DiO + DiI)(11.1%) LNPs exhibited a slight blue shift compared with those of (DiO + DiI)(11.1%) DMSO solution. Fig. 4B shows that the emission spectrum of the physical mixture exhibited both DiO emission (peak 510 nm) and DiI emission (peak 570 nm) wavelengths. Both (DiO + DiI)(11.1%) DMSO solution and physical mixture (DiO(5.9%) NPs + DiI(5.9%) NPs exhibited significant DiO-related emission (∼ 510 nm), while the FRET (DiO + DiI)(11.1%) LNPs had a sizeable emission of DiI at 570 nm without DiO-related emission at the excitation of 405 nm. Again, the slight blue shift of emission of the NPs compared to the fluorophore in solution can be contributed to the formation of H-aggregates in NPs. This will be thoroughly discussed in the next section. Overall, this analysis showed that when the LNPs were simultaneously loaded with both DiO and DiI, the resulting particles display an efficient FRET emission behaviour.

**Fig. 5:**
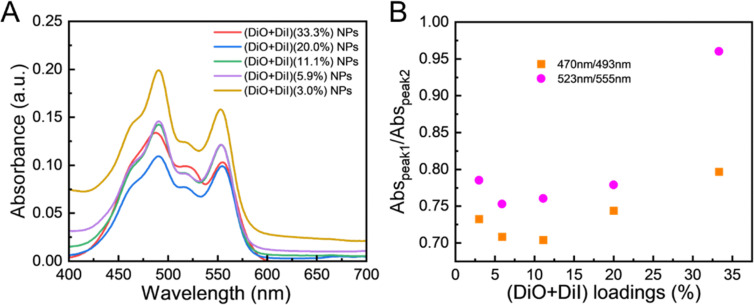
The absorbance properties of dual fluorophore loaded LNPs. (A) The absorption spectra with all LNPs diluted to the same concentration with regard to the total amount of dye, and (B) the ratios of absorbance peaks of (DiO + DiI) LNPs with different fluorophore loadings.

Both high local fluorophore concentrations and the internalisation into LNPs contribute to quenching due to aggregate formation. DiO and DiI are both carbocyanine fluorophores, which easily form intermolecular van der Waals interactions, promoting fluorophore aggregation.^65^ To understand the reason for self-quenching in this case, absorption spectra of all LNPs were recorded (Fig. 5). Overall, at high loadings the spectra become deformed and show non-linearity.^44,61,62^ All (DiO + DiI) LNPs showed two characteristic absorption peaks, as shown in Fig. 5A, which present the absorbance of DiO at 493 nm and DiI at 555 nm, respectively. Additionally, all absorption spectra were deformed and exhibited an increasing relative absorption in the region of the 0–1 vibronic shoulder at ∼ 470 nm and ∼ 523 nm, as the fraction of fluorophores increases compared to the absorption maximum. The position of the absorption band in a wavelength region (∼470 nm and ∼523 nm) shorter than that of the monomer (493 nm for DiO, 555 nm for DiI) was associated with the emissive H-aggregates.^62^ The intensity of the 0–1 vibronic shoulder relative to the intensity of the monomer peak was found to display a minimum for the LNPs with 5.9% and 11% DiO + DiI fluorophore loading. Higher fluorophore loadings resulted in an increase in the relative intensity of the 0–1 vibronic shoulder (Fig. 5B). According to the exciton theory,^66^ carbocyanine fluorophores encapsulated in LNPs stack together face-to-face driven by strong intermolecular attractive forces, which splits the excited state into two levels, but only the transition to the higher energy excited state is allowed, resulting in a blue-shifted band as compared to that of the monomer. Therefore, H-aggregate fluorophores increase demand for excitation energy, resulting in their excited states often decaying *via* non-radiative pathways to the ground state and fluorescence quenching occurring, the main mechanism of aggregation-induced quenching. Thus, regarding the absorption properties for the NPs here, the observed decreases in fluorescence and quantum yield can be associated with the formation of H-aggregates.

### The dissolution study of fluorescence-based lipid nanoparticles

To monitor the time-related dissolution behaviour of NPs, fluorescence spectra of (DiO + DiI)(11.1%) NPs were recorded with an incremental addition of DMSO, a water-miscible, good solvent for both the fluorophores and the lipid, chosen to dissolve NPs. The FRET method is sensitive to the distance between the DiO (donor molecule) and DiI (acceptor molecule) in LNPs and any dissolution of the particles should be observed as a change in the FRET behaviour.

**Fig. 6:**
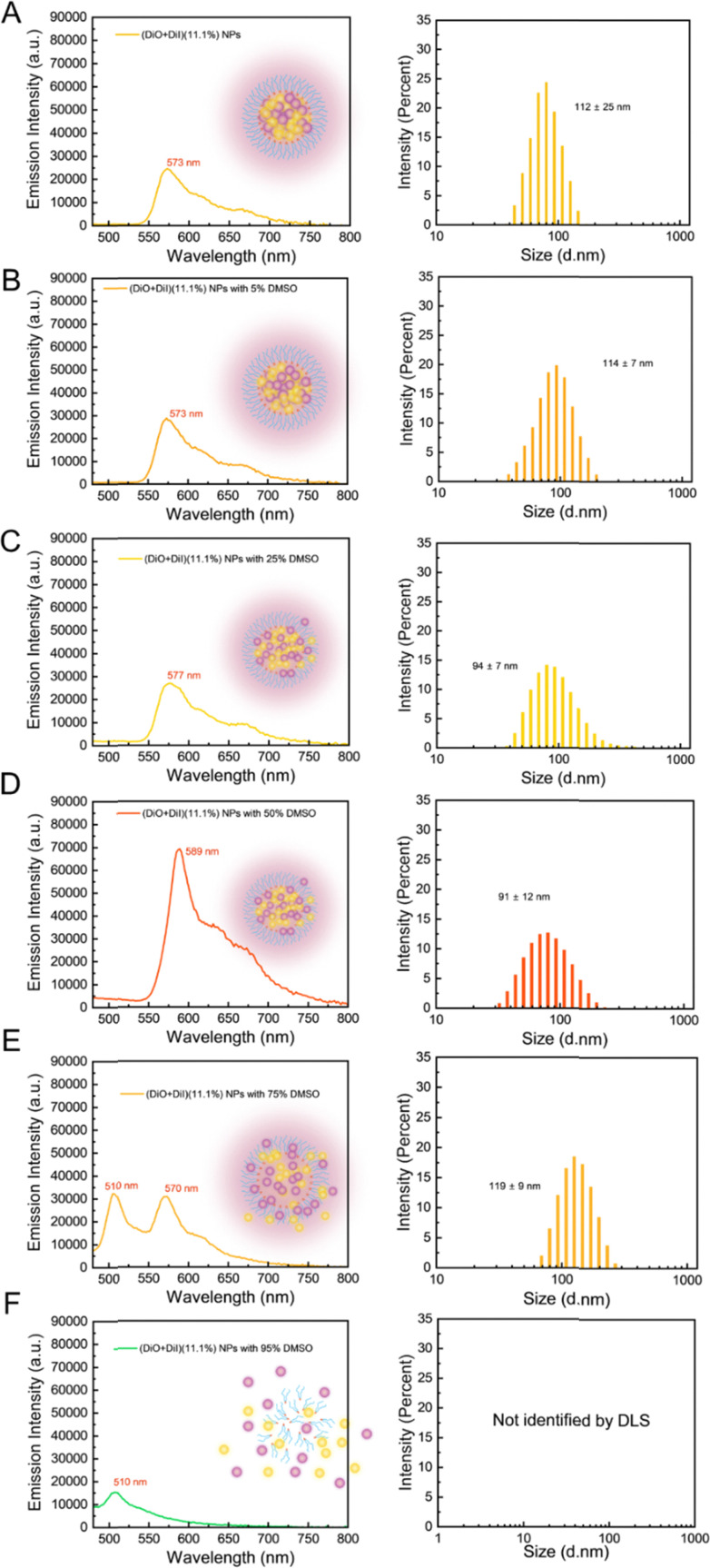
The dissociation process of dual fluorophore loaded lipid nanoparticles by the incremental addition of DMSO (5–95%). The fluorescence spectra and size distribution graphs of (DiO + DiI)(11.1%) LNPs with (A) 0% DMSO, (B) 5% DMSO, (C) 25% DMSO, (D) 50% DMSO, (E) 75% DMSO, and (F) 95% DMSO. All the emission spectra were excited at 405 nm.

Fig. 6 shows the fluorescence emission behaviour and particle size distribution of the LNPs upon the addition of DMSO, a good solvent for both the lipid and the fluorophores. In the absence of any DMSO, the LNPs showed a single emission peak at 573 nm with a diameter of 112 nm (Fig. 6A). No change in emission spectra was observed upon the addition of up to 5–25% DMSO (Fig. 6B and C). With 50% DMSO (Fig. 6D), a sizeable increase in emission intensity (∼2.5-fold) and a distinct red shift at ∼589 nm were observed (Fig. 6D). Considering the dilution effect with the additional solvent, a decrease in emission intensity would typically be expected (Fig. S9A, ESI†). We further studied the particle diameter distribution by DLS measurement. The particle diameter increased slightly with 5% DMSO and then decreased with 25–50% DMSO (trends for mean diameter and PDI can be seen in Fig. S9B, ESI†). A possible interpretation of the changes in fluorescence and particle diameter is that the addition of DMSO began to solvate the particle structure, potentially dissolving some of the fluorophores into the continuous phase. This potential dissolution of fluorophores would reduce the aggregated quenching effect, which may explain the increase of emission intensity. However, LNPs did not fully dissociate, as observed by the continued FRET signal, which means that the particles still maintained a sufficiently close environment for efficient energy transfer between DiO and DiI. With further DMSO addition, at 75% DMSO (in Fig. 6E), the emission of the DiO donor at 510 nm was observed, suggesting the dissolution of NPs where an increase in distance among DiO and DiI could no longer efficiently participate in FRET. The particle diameter also increased. Using 95% DMSO (in Fig. 6F), NPs exhibited only emission wavelength at 510 nm, which means that DiO and DiI have been solubilised and are not contained in LNPs, while the sample was not suitable for DLS analysis due to the dissolution of the particles.

**Fig. 7:**
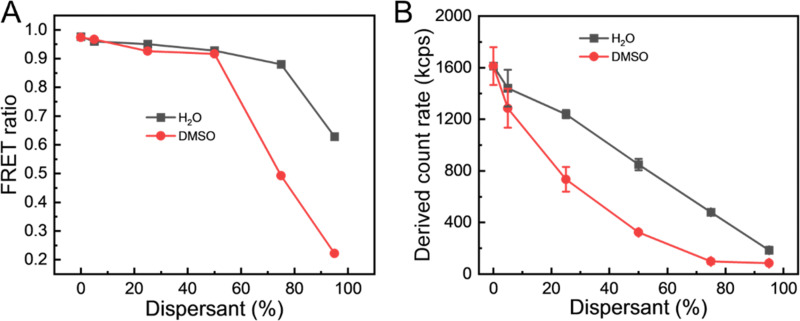
Effect of dissolution and dilution on the (DiO + DiI)(11.1%) LNPs. (A) The FRET ratios and (B) the derived count rate of the LNPs in 0% to 95% DMSO or 0% to 95% water.

Further insight into this process of particle dissolution by DMSO can be obtained by considering the FRET ratio and compared with the effect of dilution of the particles with water (a non-solvent for the lipids and fluorophores). Additionally, by considering the trends in particle diameter, PDI and the derived count rate (DCR), the integrity of the particles can be assessed. DCR is a useful measure to combine with diameter and PDI data in DLS as it provides information on the light scattering intensity of the sample.^67^ Dissolution of the particles would be expected as a decrease in the DCR as the numbers of particles are reduced and, potentially, as the difference in the refractive index between the particles and the continuous phase is reduced. In Fig. 7A, at 50% of DMSO, the FRET ratio remained still high at 0.92, further supporting that NPs had not yet dissolved completely. Upon NPs dissolution at 75% DMSO, the FRET signal decreased to 0.49 along with the increase in the donor signal due to the increased distance donor and acceptor molecules further supported by DCR (Fig. 7B). With the addition of DMSO, DCR of NPs exhibited a sharp decrease compared to the dilution experiment. At 95% of DMSO, the FRET ratio of NPs approached 0.2, which suggests that the LNPs have been dissolved and that the DiO and DiI are no longer co-located. In comparison, when diluted with water, the FRET ratio stayed constant except for the addition of 95% water, because the FRET ratio was calculated by relative emission intensities of DiI to DiO. Increasing the water content in particle solution notably resulted in a reduction of DiI emission wavelength (570 nm), while DiO emission consistently remained at a minimum. These results show that the FRET emission behaviour of the DiO + DiI LNPs can be used to report on particle integrity and dissolution.

### FRET signal photobleaching experiments *in vitro*

Next, to assess the potential of the FRET-based LNPs (DiO + DiI)(11.1%) in living cells, we analysed the FRET signals occurring by comparing the donor fluorescence intensity with cultured human cells, both inside and outside the cells. Photobleaching, a process in which the fluorophore is destroyed by prolonged excitation, resulting in a permanent loss of its fluorescence properties,^68^ was used to investigate the changes in the FRET emission within the LNPs by selective irradiation of the DiI fluorophore using 50 iterations of 561 nm laser light. Upon comparison of fluorescence intensity in the same region before and after photobleaching the acceptor, if the FRET signal is present in the intact nanoparticles, the destruction of the DiI molecule will induce a distinct increase in donor (DiO) fluorescence intensity.^69^ This is because in the absence of FRET, the donor fluorescence intensity is no longer quenched by the acceptor, thus showing a clear donor signal increase, which directly reveals the energy transfer occurring from donor molecule.

**Fig. 8:**
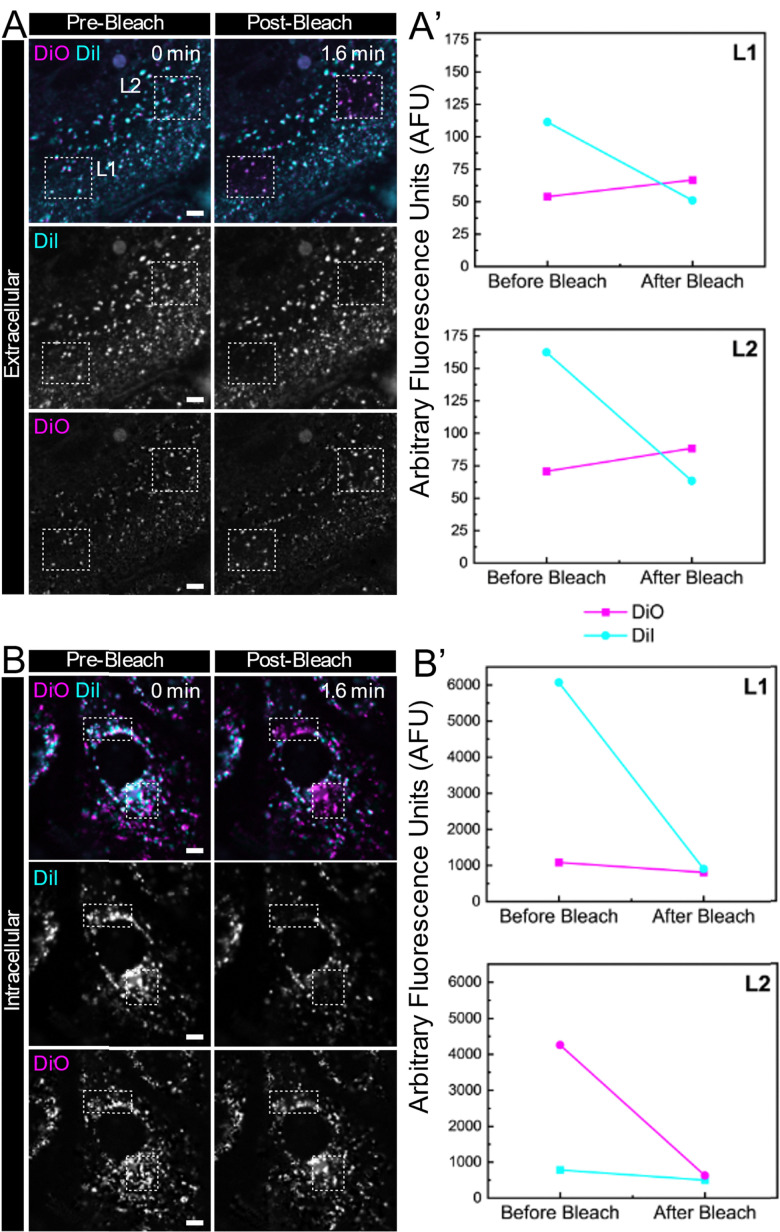
FRET assay between the donor (DiO) and acceptor (Dil) for the (DiO + DiI)(11.1%) LNPs. Cells were incubated with (DiO + DiI)(11.1%) LNPs and imaged before and after photobleaching the acceptor molecule (DiI) using 561 nm laser light in the defined areas (hashed lines) both before internalisation (A) and after internalisation (B). Corresponding fluorescence intensities of the donor (DiO) and acceptor (DiI) were recorded for two areas of interest (L1 and L2) before internalisation (A′) and after internalisation (B′). The scale bar corresponds to 5 μm.

For regions outside of the cells (Fig. 8A), upon the excitation of 488 nm (selected based on the suitable excitation wavelengths available for confocal microscopy), a strong extracellular DiI emission intensity and a minimal DiO emission intensity were observed in both two regions of interest, L1 and L2. Upon the selective photobleaching of DiI, a clear increase in DiO emission intensity was observed (Fig. 8A′), revealing the disruption of the energy transfer process between the DiO and DiI, further validating the integrity of the (DiO + DiI)(11.1%) LNPs inside cells. When compared to fluorescent signals obtained associated with LNPs that have undergone cellular internalisation (Fig. 8B), there was a drop of DiI emission intensity after laser bleaching in regions which was not accomplished by an increase in DiO emission, indicating the disruption of FRET (Fig. 8B′). The lack of a FRET signal following internalisation indicates that the nanoparticles had dissociated, impeding the efficiency of energy transfer between the DiO and DiI. The selective photobleaching acceptor is a quite straightforward way to illustrate the dynamic process of nanoparticles by the FRET signal, confirming that FRET occurs primarily in intact nanoparticles outside the cells and disappears as nanoparticles disintegrate inside the cells. This approach offers a distinct advantage in this case where the increase of donor intensity observed in regions with a photobleached acceptor corresponds to intact nanoparticles, reinforcing the value of this technique for visualising real-time signalling events *in vitro* response to the transition of nanoparticles from an intact to a dissociated state. Our future work will build on these proof-of-concept *in vitro* studies to more fully understand the kinetics of particle dissolution upon internalisation.

## Conclusion

A comprehensive study of DiO and DiI photophysical behaviours in the LNPs environment has been presented. We investigated the FRET behaviour of fluorescence-based LNPs and the effect of high fluorophore loadings on their characteristics. We first optimised the formulation of LNPs loaded with a FRET pair produced by nanoprecipitation.

Although high local fluorophore concentration led to aggregation-induced quenching, observed by distinct quenching, blue shift and broadening of fluorescence spectra due to H-aggregate formation, our system achieves an optimal FRET efficiency of 0.98 at 11.1% fluorophore loading, higher than any previously reported for FRET LNPs. As such our system balances signal integrity and minimal quenching, an important consideration in the design of FRET-based LNPs. The quantum yields of all formulations were three times those of pure fluorophores in ethanol.

The dissolution behaviour of the FRET LNPs was studied in detail to reveal three distinct structural transitions, such as solvation, swelling and partial dissolution, and then finally complete dissolution. This represents a significant advance in correlating fluorescence signals with real-time structural changes, providing a new dimension to nanoparticle stability and release studies. Furthermore, *in vitro* cell experiments demonstrated proof of concept of the utility of the FRET LNPs. An acceptor photobleaching experiment was used to confirm the correlation between FRET occurrence and nanoparticle integrity, which provided clear evidence of FRET signalling in intact nanoparticles outside cells and a loss of this signal following nanoparticle disintegration inside the cells.

FRET-enabled LNPs are potentially a powerful tool for studying nanoparticle integrity, with implications far beyond traditional drug delivery. By enabling the real-time monitoring of structural changes in complex settings, this platform paves the way for applications in diagnostics, targeted delivery, and the rational design of next generation nanocarriers. Using this technique, researchers can effectively track the integrity and behaviour of nanoparticles, making it an ideal tool for studying dynamic processes such as cellular uptake, drug release, or molecular interactions in real-time, suggesting broader applications in studying LNP-based delivery systems.

## Data availability

The data supporting this article have been included as part of the ESI.†

## Conflicts of interest

The authors declare no conflicts of interest.

## Supplementary Material

TB-013-D4TB02653E-s001
