# Supplementary material for: Capturing the dynamic integrity of carbocyanine fluorophore-based lipid nanoparticles using the FRET technique
Source: J Mater Chem B. 2025 Jan 22;13(7):2295–305. doi: 10.1039/d4tb02653e (PMC11783621; doi:10.1039/d4tb02653e)
Supplement: TB-013-D4TB02653E-s001 [file TB-013-D4TB02653E-s001.pdf]

Supplementary Information (SI) for Journal of Materials Chemistry B.
This journal is © The Royal Society of Chemistry 2025

SUPPLEMENTARY INFORMATION

Tabel S1. All formulations with individual components represented by weight (mg)

Formulations	Lipid content (mg)	Fluorophores (mg)	
		DiO	DiI
DiO (11.1%) DMSO	/	0.075	/
DiI (11.1%) DMSO	/	/	0.075
Pure LNPs	0.6	/	/
DiO (5.9%) NPs	0.6	0.0375	/
DiI (5.9%) NPs	0.6	/	0.0375
(DiO+DiI)(3%) NPs	0.6	0.009375	0.009375
(DiO+DiI)(5.9%) NPs	0.6	0.01875	0.01875
(DiO+DiI)(11.1%) NPs	0.6	0.0375	0.0375
(DiO+DiI)(20%) NPs	0.6	0.075	0.075
(DiO+DiI)(33.3%) NPs	0.6	0.15	0.15

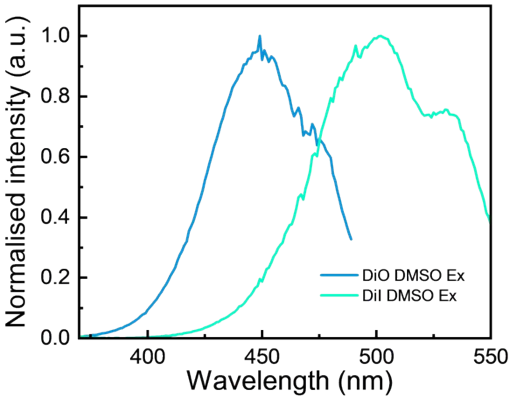

Figure S1. The normalised excitation intensity of DiO(11.1%) DMSO at emission of 510 nm and DiI(11.1%) DMSO solutions at emission of 580 nm.

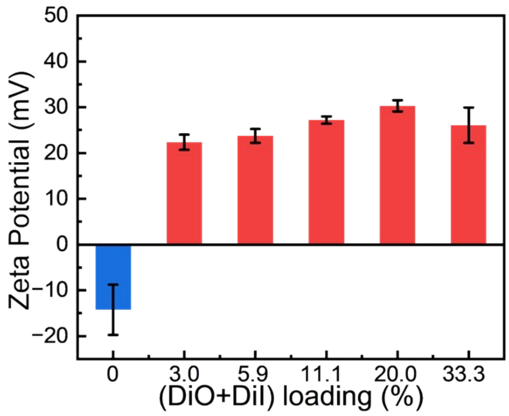

Figure S2. The Zeta potentials of dual fluorophore loaded lipid nanoparticles with different loadings.

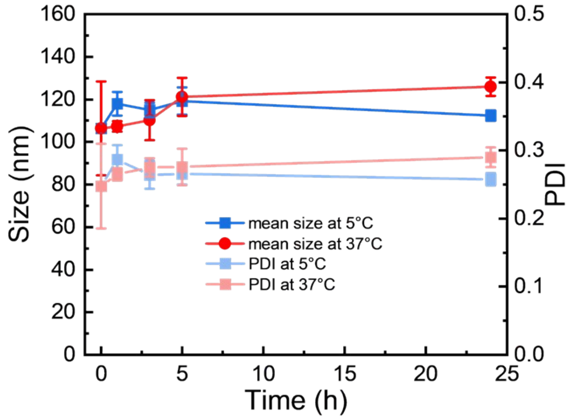

Figure S3. The hydrated sizes of (DiO+DiI)(11.1%) NPs at 5°C and 37°C over 24 hours by DLS measurement.

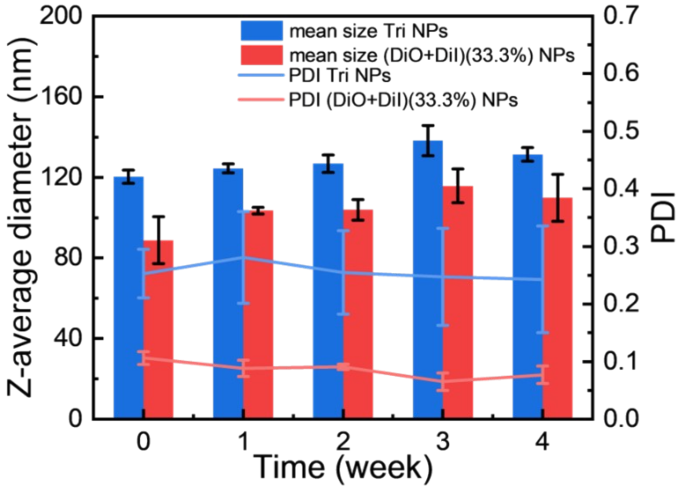

Figure S4. The hydrated sizes of nanoparticles (without and with fluorophores) at 5°C within 4 weeks by DLS measurement.

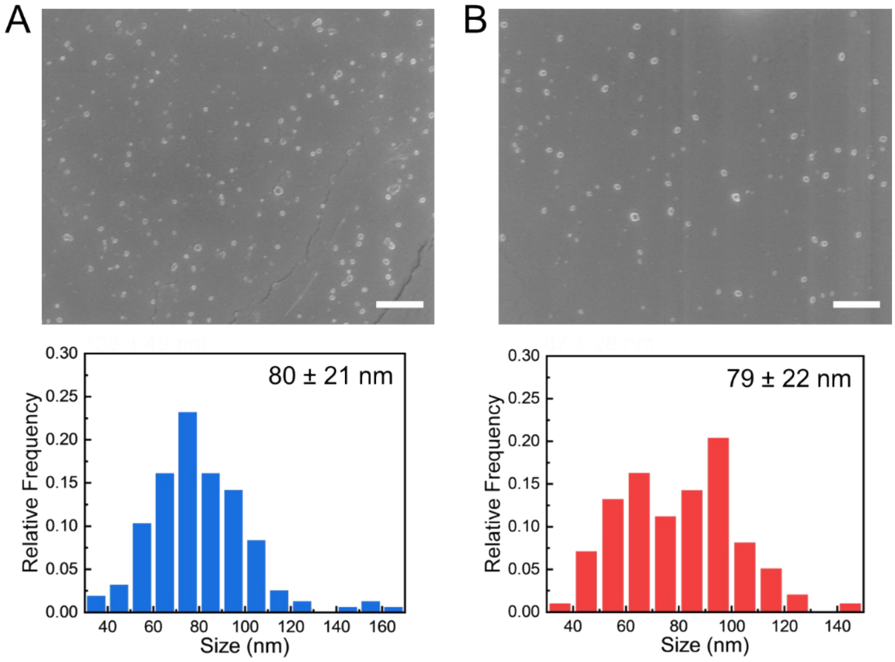

Figure S5. SEM images and their particle size distribution of A: pure lipid nanoparticles and B: (DiO+DiI)(11.1%) lipid nanoparticles. The scale bar is 1 μm.

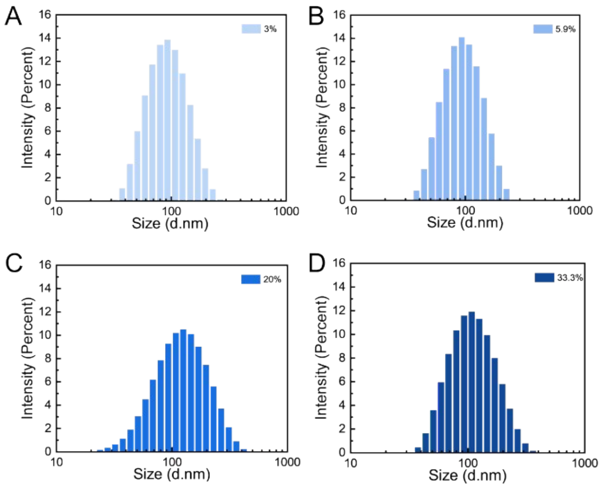

Figure S6. The size distribution graphs of dual fluorophore loaded lipid nanoparticles with different loadings by DLS measurement. A: (DiO+DiI)(3%) NPs. B: (DiO+DiI)(5.9%) NPs. C: (DiO+DiI)(20%) NPs. D: (DiO+DiI)(33.3%) NPs.

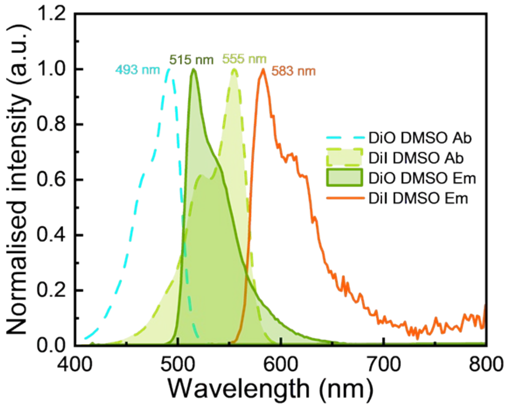

Figure S7. Absorbance and fluorescence spectra of DiO(11.1%) DMSO and DiI(11.1%) DMSO solution. Fluorescence spectra were collected under 405 nm of excitation. The shading represents overlap between the emission spectrum of DiO (11.1%) DMSO and the absorption spectrum of DiI (11.1%) DMSO.

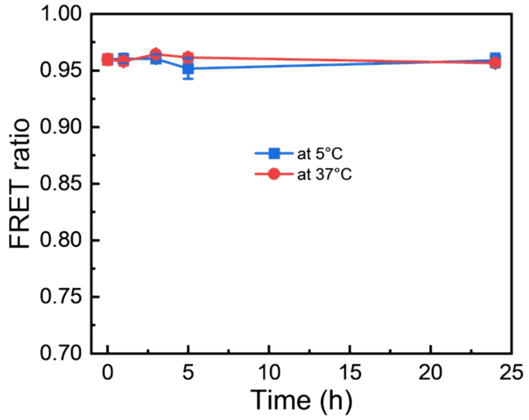

Figure S8. FRET ratio of (DiO+DiI)(11.1%) NPs at 5°C and 37°C over 24 hours.

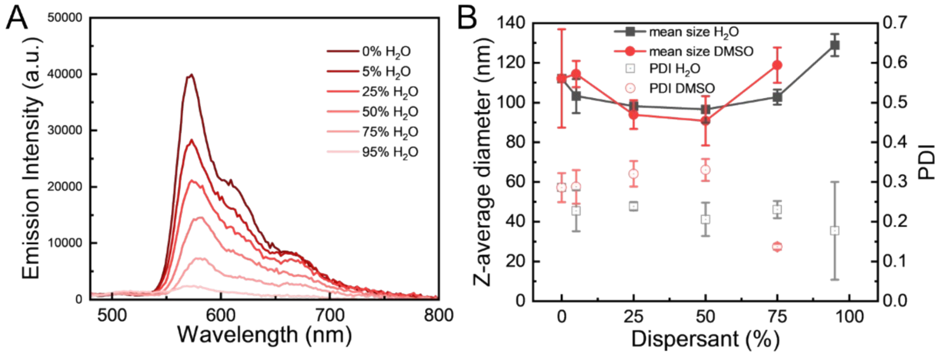

Figure S9. A: Fluorescence spectra at excitation of 405 nm and B: the diameter of (DiO+DiI)(11.1%) NPs with addition of water and DMSO.
